# Headaches and Headache Cures

**Published:** 1907-02-09

**Authors:** 


### HEADACHES AND HEADACHE CURES.

In view of the frequent occurrence of headache as a troublesome symptom among all classes of the community, it is not surprising that the manufacturers of proprietary medicines have seen the commercial opportunities offered by a number of the recently introduced analgesics. These, under various attractive titles, are now being sold in considerable quantities, and their efficacy is in many instances entirely beyond dispute. Headache, it is true, may be the early expression of some serious disease. But it is frequently a mere functional disturbance, and thus it is not a matter for surprise that remedies which relieve it are eagerly purchased. The medical profession has many reasons to suspect the pretensions of the advertising chemist; but this suspicion is carried too far when it leads to a determination to refuse to prescribe everything proposed as a new remedy. On the contrary, it is not less the duty than the interest of the profession to keep an open mind towards new developments, and to study the exact conditions under which new forms of treatment can be safely and successfully prescribed. To aid in this direction it is here proposed to give some account of "headache cures," of the degree of their success, and of the extent to which their use involves secondary and undesirable consequences.

The chief groups of drugs used for the relief of headache *per se* are represented by the bromides, chloral, phenacetin, phenazone, acetanilide, and caffeine.

### Bromides.

Of the potassium, sodium, and ammonium salts of bromine but little need be said; their action is well known and their disadvantages are generally appreciated. The depressant effect of the bromides, particularly if administered frequently and regularly, is the chief objection to their habitual use for migraine, and the relief derived from them does not leave the mental faculties sufficiently clear for the transaction of daily affairs, as do many of the other remedies. And it should be recollected in prescribing proprietary medicines of unknown composition, that in order to ensure the more prompt and unfailing working of a somewhat undependable drug, bromides are often mixed or combined with the quack compounds and the fact disguised under a registered trade-name. Such remedies as *bromipin*, *brominol*, and *brominoleum*, denote by their titles the active principle in their composition, but they give no indication of the proportions present in the mixture.

### Chloral.

The basis upon which all the chloral compounds depend is chloral hydrate—a powerful, unvarying, and dangerous hypnotic; of the utmost value in skilled hands, peculiarly attractive for self-administration, and exceedingly fatal in an overdose, since the tolerance acquired in the habitual use of morphine does not appear to be established with chloral.

The official preparations are two only: chloral hydrate (dose, 5-20 grains) and the syrup, containing 10 grains in a drachm (dose, ½-2 fluid drachms). The non-official remedies into whose composition chloral enters are innumerable:—

*Bromidia*, combining chloral hydrate with one or other of the bromides and extracts of cannabis indica and hyoscyamus.

*Dormiol* (amylene chloral).

*Chloralamide*, a compound of chloral anhydride and formamide, said to have a less depressant influence on the heart, and to retain its efficacy after prolonged administration.

*Chlorobrom*, a mixture of chloralamide with potassium bromide, potent—valuable, and dangerous.

*Somnal*, a mixture of chloral with urethrane.

*Chloralose* (anhydro-glyco-chloral), more toxic even than chloral hydrate, rapidly ceasing to be effective as the patient becomes accustomed to the drug, and, generally speaking, not worth the risk of administration for the relief of migraine.

Allied to chloral hydrate we have also:

*Butyl chloral hydrate* (*syn.* croton chloral hydrate), a drug the efficacy of which is undoubted in some cases of neuralgia, especially of the fifth nerve, but its occasional successes seem less numerous than its frequent disappointing failures.

*Chloretone* (tri-chlor-butyl-alcohol) possesses practically no advantages over the plain chloral hydrate, and the additional disadvantage that its therapeutic effect and toxicity are alike more variable. The known toxicity of chloral hydrate constitutes to some extent a safeguard in its administration by skilled hands.

### Phenacetin.

This represents possibly the safest group of drugs for "headache"; the after-effects are slight, the mental state is undimmed by the administration, and use does not seem to give rise to habit-formation; possibly there is a tendency to resort to alcoholic "pick-me-ups" after repeated doses at short intervals. The name "phenacetin" has in itself no meaning, and bears no relation to the actual chemical composition which is that of a paraphenetidin compound with acetic acid.

The activity of this class depends upon the paraphenetidin molecule and the strength of the various members of the phenacetin class can be estimated, as it were, upon this basis. Besides phenacetin itself, the only preparation worthy of note is the *para-phenetidin lactate* (usually known as "lactophen"), which possesses in an even more marked degree the sedative powers of its ally, is dependable, and has an unvarying chemical composition; it is insoluble, and the dose is given as 5-15 grains.

Apart from this compound, most of the phenacetin derivatives are either mixtures or such loosely combined chemical substances that they decompose readily and thereby become utterly unreliable.

*Apolysin* (monobasic paraphenetidin citrate), *citrophen* (dibasic paraphenetidin citrate), *kephaldol* (a strange medley of paraphenetidin citrate and quinine, probably a mere mixture and certainly inert), for which many things have been claimed. *Malakin* (paraphenetidin salicylate). *Phenosal* (paraphenetidin acetosalicylate). *Kryofin* (paraphenetidin acetoglycollate); and so on until the whole gamut has been sounded of organic salts of paraphenetidin, for all of which the claims become daily more impudent and the mis-statements more unblushing. To sum up, it may be said that the paraphenetidin salts have each and all some effect as hypnotics, sedatives, and analgesics, but that the acid-radicle with which the paraphenetidin molecule is combined has no further action than to dilute the effect of the compound according to the proportion it bears to the total molecular weight.

### Phenazone, or antipyrin, ranks perhaps next after phenacetin for safety and absence of ill-effects; there seems little or no tendency to habit-formation with the simple drug, but many of the derivatives on the market contain less innocuous elements, and these will be referred to in their place. By itself antipyrin exerts a rapid and useful effect upon many headaches of the migrainous character, but repeated doses may have a cumulative action and produce alarming toxic symptoms. Dose, 5-20 grains, readily soluble in water; it is incompatible with many other drugs and is usually best administered alone. All that has been said of the paraphenetidin salts may be applied with no less force to the antipyrin group. The "fancy" compounds are in brief: *Aceto-pyrine* (antipyrin aceto-salicylate), also called pyrosal; *salipyrin* (antipyrin salicylate); *tolypyrin*, a chemical ally of antipyrin whose composition is doubtful, but its non-activity established, from which has been derived *tolysal*, the salicylate of tolypyrin; *ferripyrin*, a compound of ferric chloride and antipyrin, possessing a colour pleasing to the eye and apparently harmless to the system; *bromopyrin*, a mixture or compound presenting the combined effects of uncertain proportions of antipyrin and a bromide; *hypnal*, a compound with chloral which should be properly included under its more potent and dangerous constituent; chloral hydrate should be preferred to hypnal as a drug whose action is known and toxicity well established. *Pyramidon* (dimethyl-amido-antipyrin), together with its two *camphorates* and a *salicylate*, may possess an antipyretic effect, but is therapeutically devoid of value as an analgesic or sedative.

### Acetanilide.

Acetanilide, or antifebrin, forms the third of the group of synthetic compounds upon which the majority of "headache cures" are based. It is more powerful, both in its therapeutic action and its toxic effects than either phenacetin or antipyrin. The dose is 1 to 3 grains. (N.B.—60 grains taken in six powders have proved fatal).

Acetanilide enters largely into certain proprietary drugs. *Antikamnia*, an unknown combination probably containing acetanilide, caffeine, and sodium bicarbonate; *antinervine*, consisting of acetanilide, ammonium bromide, and salicylic acid; *ammonol* and *phenalgin* are ammoniated derivatives of acetanilide; *antisepsine*, although not betraying by its title any connection with acetanilide, depends for its action on the fact that this enters largely into its composition; while the same is true of the speciality known as *antitoxine*. *Bromo-acetanilide*, a so-called compound of bromine with acetanilide, may be to all intents regarded as a mixture of acetanilide with a bromide, even when masquerading under its pseudonyms of *antisepsin* and *asepsin*. *Hydracetin* is a derivative of acetanilide, chemically known as acetyl-phenyl-hydrazin, more powerful and dangerous even than the former. *Exalgin* (methyl acetanilide) possesses no advantages over acetanilide, but may be used as a substitute, for its action is practically identical, though both action and toxicity are greater. Dose, ½ to 1 grain.

### Caffeine.

Apart from the foregoing groups of drugs caffeine enters so commonly into the composition of proprietary "headache cures" that it deserves some mention. Taken by itself it is only occasionally effective, but in combination with phenacetin or antipyrin it appears to aid in their action and perhaps counteracts their possible depressant effects. The majority of so-called caffeine compounds, such as caffeine hydrobromide, are so unstable as to decompose in the presence of water, thereby being in no way better than a mixture of the constituents, which are cheaper and more readily estimated as therapeutic agents. *Migramine* is the fancy name for a double citrate of caffeine and antipyrin. *Antikamnia* has been already mentioned as a compound of acetanilide with caffeine, while *pyretine* and *phenatol* are suspected of owning to the same active ingredients.

From the above it will be readily seen that by combining the ordinary pharmacopœial drugs into such mixtures as experience may suggest, no less reliable compounds may be arrived at than those so loudly vaunted by the patent-medicine vendors, and the satisfaction of knowing beforehand the proportions and probable therapeutic effects of the individual drugs, whose respective actions remain usually unaltered by mixing, should far outweigh the mere elegance of a title assumed to give an air of "verisimilitude to an otherwise bald and unconvincing narrative" of quackery.

